# Associations of Amino Acids with the Risk of Prediabetes: A Case-Control Study from Kazakhstan

**DOI:** 10.3390/jpm14101067

**Published:** 2024-10-21

**Authors:** Alma Nurtazina, Ivan Voitsekhovskiy, Bakyt Kanapiyanov, Maxat Toishimanov, Daulet Dautov, Kairat Karibayev, Yerbol Smail, Dana Kozhakhmetova, Altay Dyussupov

**Affiliations:** 1 Department of Epidemiology and Biostatistics, Semey Medical University, Semey 071400, Kazakhstan; alma.nurtazina@smu.edu.kz; 2 Outpatient Clinic #1, Department of Internal Medicine and Cardiology, Semey Medical University, Semey 071400, Kazakhstan; 3 Faculty of Biology and Biotechnology, Al-Farabi Kazakh National University, Almaty 050038, Kazakhstan; 4 Department of Propaedeutics of Internal Diseases, Semey Medical University, Semey 071400, Kazakhstan; bakyt.kanapiyanov@smu.edu.kz; 5 Food and Environment Safety Laboratory, Kazakstan-Japan Innovative Center, Kazakh National Agrarian Research University, Almaty 050010, Kazakhstan; maxat.toishimanov@gmail.com; 6 Department of Propaedeutics of Internal Diseases, Asfendiyarov Kazakh National Medical University, Almaty 050012, Kazakhstan; dautov.d@kaznmu.kz; 7 Central Clinical Hospital, Almaty 050040, Kazakhstan; karibayev@ckb.kz; 8 Department of Infectious Diseases, Dermatology and Immunology, Semey Medical University, Semey 071400, Kazakhstan; erbol.smail@smu.edu.kz; 9 Department of Internal Diseases, Semey Medical University, Semey 071400, Kazakhstan; dana.kozhakhmetova@smu.edu.kz; 10 Rector Office, Semey Medical University, Semey 071400, Kazakhstan; altaidusupov@gmail.com

**Keywords:** prediabetes, amino acids, alanine, valine, prediction, Kazakh population

## Abstract

Background: The high global prevalence of prediabetes requires its early identification. Amino acids (AAs) have emerged as potential predictors of prediabetes. This study investigates the association between amino acids and prediabetes in the Kazakh population. Materials and Methods: In this case-control study, serum AAs levels were measured using the Trace GC 1310 gas chromatography system coupled with the TSQ 8000 triple quadrupole mass spectrometer (Thermo Scientific, Austin, TX, USA) followed by silylation with the BSTFA + 1% TMCS derivatization method. Biochemical parameters, including total cholesterol, HDL-C, LDL-C, triglycerides, fasting glucose, HbA1c, and Creatinine, were assessed for each participant. Trained professionals conducted anthropometric and physical examinations (which included taking blood pressure and heart rate measurements) and family history collection. Results: A total of 112 Kazakh individuals with prediabetes and 55 without prediabetes, aged 36–65 years, were included in the study. Only Alanine and valine showed a significant association with prediabetes risk among the 13 AAs analyzed. Our findings revealed an inverse relationship between Alanine and Valine and prediabetes in individuals of Kazakh ethnicity. Conclusion: A lower serum level of Alanine and Valine may serve as a predictive biomarker for prediabetes in the Kazakh population.

## 1. Introduction

The global prevalence of prediabetes and type 2 diabetes mellitus (T2DM) has reached epidemic proportions, particularly among non-white ethnic groups [1]. Prediabetes, as an intermediary state, significantly heightens the risk of progressing to T2DM, metabolic syndrome (MS), chronic kidney disease (CKD), and cardiovascular complications [2]. Therefore, early detection of prediabetes is crucial for implementing preventive measures that can mitigate its advancement to diabetes with clinical manifestations. Currently, clinical settings utilize five distinct definitions of prediabetes, each articulated by varying thresholds of hemoglobin A1c (HbA1c), fasting glucose, and 2-h glucose measurements [3]. This divergence has prompted criticism surrounding the nomenclature of “prediabetes”, as it conveys a sense of inevitability regarding the onset of diabetes; however, it has been observed that approximately one-third of individuals with prediabetes experience a return to normal glycemic levels over time [4,5]. In light of these considerations, the World Health Organization (WHO) has advocated for the adoption of the term “intermediate hyperglycemia” as a more accurate descriptor of this condition [6].

Patients with prediabetes who also exhibit MS are characterized by heightened incidences of hypertension, dyslipidemia, and abdominal and metabolic obesity [7]. Alterations in metabolite levels in prediabetes may serve as biomarkers or predictors of prediabetes risk, and thereby enable early, effective preventive measures. Emerging research suggests that AAs may serve as pivotal predictors of prediabetes [8,9]. Studies have demonstrated that individuals with prediabetes frequently present impaired kinetics and concentrations of Valine and Alanine, indicating disturbances in the metabolism of AAs that could be integral to the condition’s pathophysiology [10,11]. Furthermore, experimental studies utilizing HepG2 cell lines have indicated that branched-chain amino acids (BCAAs) supplementation can influence glucose and lipid metabolism, potentially diminishing hepatic lipogenesis and gluconeogenesis—key processes involved in the development of insulin resistance (IR) and fatty liver disease [12].

Ethnic and racial variations in the predictive capacity of AAs for T2DM have been increasingly acknowledged in current research [13]. Individuals identified as Asian, Black, or Hispanic exhibit an elevated likelihood of prediabetes, as well as those possessing lower educational attainment [14,15]. Notably, metabolic biomarkers—including BCAAs, aromatic AAs, glycoprotein acetyls, total fatty acids, and triglycerides—have been consistently linked to an elevated risk of T2DM among various Asian ethnic groups [16]. However, there remains a significant gap in the literature regarding AAs’ profiles in Central Asian populations and their relationship to prediabetes. This study aims to address this knowledge gap by investigating the association between AAs and prediabetes, thereby contributing to a more nuanced understanding of metabolic risk factors within the Kazakh population.

## 2. Materials and Methods

This case-control study was conducted at five primary care centers (PHCs) in Semey City, Abay Oblast, East Kazakhstan. We recruited participants between December 2022 and March 2024.

### 2.1. Sampling

We used two-stage sampling for the selection of participants. In the first stage, we randomly selected five out of 40 general practices (GPs) in Semey City. Following this, sample frames were established for potential participants corresponding to each selected GP from lists of patients meeting the predetermined inclusion criteria. In the second stage, 50 participants were randomly selected from each GP unit. Randomization was conducted using computer software to generate random numbers.

### 2.2. Data Collection and Measurement

Standardized questionnaires were employed to gather demographic information, including smoking status, family history, and hereditary predispositions to cardiovascular diseases (CVD) and hypertension (HT). A comprehensive physical examination was conducted to assess anthropometric parameters, which included measurements of weight, height, waist circumference, and blood pressure, performed in accordance with the guidelines established by WHO, the European Society of Cardiology (ESC), and the European Society of Hypertension (ESH) [17]. A standardized stadiometer was used for height assessment and a calibrated scale for weight measurement. Blood pressure (BP) was measured utilizing the Korotkov method, following the ESH/ESC algorithm, with participants resting in a seated position during the assessment. Two consecutive measurements were taken for each participant, and the average of these values was recorded. Body mass index (BMI) was calculated using the formula: weight (kg) divided by height (m^2^). Information regarding the history of comorbidities and any medications taken was obtained from medical records and through participant interviews. All information collected for each participant was compiled into an individualized file with coded personal data to ensure confidentiality.

### 2.3. Participants

Initially, a total of 253 candidates of Kazakh ethnicity were enrolled in the study, comprising 130 individuals diagnosed with prediabetes (cases) and 123 individuals with normal glycemia (controls) based on a simple random sampling.

Exclusion criteria included ethnicity other than Kazakh, the presence of confirmed diabetes, a history of stroke or myocardial infarction (MI), heart failure, thyrotoxicosis, hypothyroidism, and the use of statin therapy for less than six months prior to the initiation of the study.

**Figure 1:**
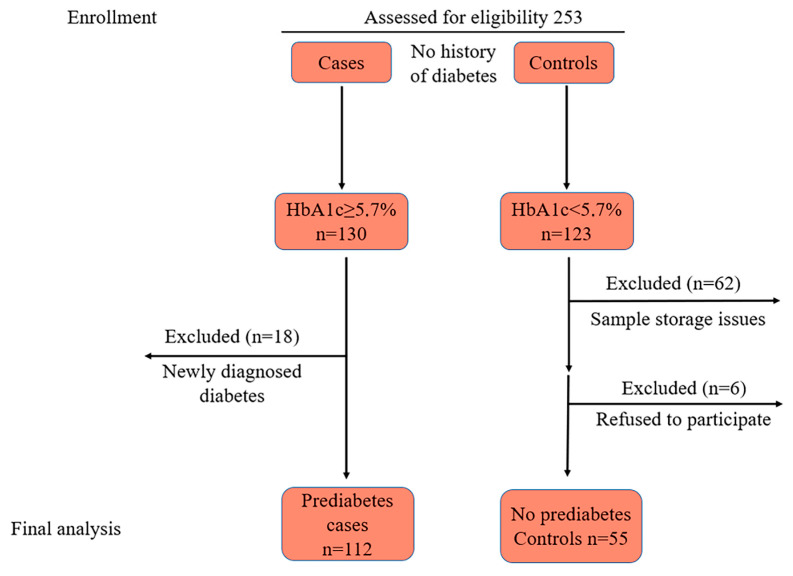
Flow diagram of participant selection.

During the laboratory phase, 62 samples were excluded due to storage issues. Additionally, at the data analysis stage, 24 participants were excluded following the diagnosis of new-onset T2DM. Consequently, data from 167 participants, 112 cases, and 55 controls were included in the final analysis (Figure 1).

### 2.4. Ethics Considerations

The research protocol received approval from the Ethics Committee of Semey Medical University (minutes #7, date of approval: 16 March 2022). In adherence to ethical standards, confidentiality and privacy were rigorously maintained throughout the study. All personal data were anonymized and stored in a secure database, accessible solely to the project manager and two designated research team members. Before participation, all individuals were provided with comprehensive information regarding the aims and objectives of the study. Participants were assured that their involvement was entirely voluntary, with the provision to withdraw from the study at any point without the requirement to provide a rationale and without any associated penalties. Following this, informed consent was obtained from individuals who met the established inclusion criteria.

### 2.5. Variables

The main outcome of interest was prediabetes. The main exposures were ta AAs, including Lysine, Tyrosine, Alanine, Valine, Leucine, Isoleucine, Proline, Serine, Threonine, Methionine, Aspartic acid, Glutamic acid, and Phenylalanine. All other factors like gender, age, history of cardiovascular diseases (CVDs) and HT/antihypertensive therapy, smoking, Creatinine, low-density lipoprotein cholesterol (LDL-C), high-density lipoprotein cholesterol (HDL-C), plasma triglycerides (TG), total cholesterol (TC), blood pressure (BP), body mass index (BMI), and waist circumference (WC), were considered as potential confounding factors. We also used MS as a binary variable to determine the proportion of those with MS.

### 2.6. Diagnostic Criteria

Dysglycemia, encompassing impaired fasting glucose (IFG), impaired glucose tolerance (IGT), prediabetes, and T2DM, was defined in accordance with the current guidelines established by the American Diabetes Association (ADA) [18]. Glycemic status was classified based on HbA1c levels, with T2DMindicated by values ≥6.5%, and prediabetes defined by values ranging from 5.7% to 6.4%. MS was diagnosed following the criteria outlined by the International Diabetes Federation (IDF) [19,20], which requires the presence of abdominal obesity along with at least two of the following four clinical factors: systolic BP > 130 mmHg or diastolic BP > 85 mmHg, serum triglycerides (TG) > 1.7 mmol/L, HDL-C < 1.03 mmol/L in men and <1.29 mmol/L in women, and serum glucose levels > 5.6 mmol/L.

Obesity was classified according to WHO criteria based on BMI, with the following categories established: normal weight as <25 kg/m^2^, overweight as 25–29.9 kg/m^2^, and obesity as >30 kg/m^2^.

### 2.7. Chemicals

#### 2.7.1. Biochemistry

All blood samples were collected in the morning following a fasting period of at least twelve hours, utilizing intravenous venesection. The levels of TC, LDL-C, HDL-C, TG, fasting glucose, HbA1c, and Creatinine were quantified in the laboratory. The AAs’ concentrations were determined via gas chromatography–mass spectrometry (GC–MS). The targeted amino acids included Lysine, Tyrosine, Alanine, Valine, Leucine, Isoleucine, Proline, Serine, Threonine, Methionine, Aspartic acid, Glutamic acid, and Phenylalanine.

#### 2.7.2. Gas Chromatography–Mass Spectrometry

High-performance liquid chromatography (HPLC) grade methanol was purchased from Sigma-Aldrich (St. Louis, MO, USA). BSTFA + 1% TMCS (N, O-bis (trimethylsilyl) trifluoroacetamide with 1% trimethylchlorosilane for GC) (>99.0% purity), methoxyamine hydrochloride (>98.0% purity), and pyridine (>99.8% purity). AAS 18 mixed standard amino acid solution (Sigma-Aldrich, St. Louis, MO, USA).

#### 2.7.3. Sample Preparation

Each 100 μL blood serum sample was mixed with 400 μL methanol–acetone mixture (2:1, *v*:*v*) to precipitate the protein. Next, the mixture was vortex-mixed for 30 s and centrifuged for 10 min (15,000 rpm) at 4 °C. The 300 μL supernatant was transferred into a 2 mL Eppendorf centrifugation tube and evaporated to dryness by N_2_ gas with a sample concentrator (Miulab, NDK200-1N, Hangzhou, China). Then 100 μL methoxamine hydrochloride in pyridine (10 mg/mL) was added to the dried tube, and the mixture was mixed on a vortex for 30 s and incubated for two hours at 37 °C. Finally, 100 μL of BSTFA derivatization agent was added to the mixture, vortexed for 30 s, and heated at 50 °C for two hours. The final solution was taken for GC–MS analysis [21,22].

#### 2.7.4. GC–MS Analysis

The plasma amino acid analysis was conducted on a Trace GC 1310 gas chromatography instrument coupled to a TSQ 8000 triple quadrupole mass spectrometer (Thermo Scientific, Austin, TX, USA) and equipped with an autosampler AS 1310. The column used for all analyses was a Thermo Scientific TG-5SilMS column (30 m × 0.25 mm × 0.25 μm). The column temperature procedure was designed as follows: initially maintained at 50 °C for 5 min, programmed to 250 °C at a rate of 5 °C/min, and then held at 250 °C for 15 min. 99% pure helium was used as a carrier gas, and the device was equipped with a triple helium gas filter (Thermo, Singapore) with a flow rate of 1.0 mL/min. The septum purge was constantly switched on with a 3 mL/min flow rate. The injector temperature was set at 280 °C, the MS transfer line temperature was set at 250 °C, and the ion source temperature was 240 °C. The mass spectrometer was operated under electron ionization (EI) in full scan mode range varied from *m*/*z* 30 to 550 with a 0.2 s scan velocity, and the detector voltage was 0.96 kV. Ionization was achieved by a 70 eV electron beam. Instrument control, data acquisition, and data processing were performed by XCalibur software, version 4.3 (Thermo Scientific, Austin, TX, USA). All the detected peak features were identified by standards and the NIST Mass Spectral Search Program [23].

#### 2.7.5. Bias

A two-stage random sampling strategy was employed to minimize selection bias, ensuring that the study population was representative of the target group. Standardized procedures were used to measure BP, height, weight, and WC, all conducted by trained personnel to reduce measurement error. Laboratory analyses were centralized in a single facility. Interviews were carried out consistently by specially trained staff to limit observer bias. Prediabetes, HT, and obesity were defined and diagnosed in accordance with pre-established criteria before the initiation of the study. The final dataset underwent independent double verification by two research team members to prevent systematic data entry errors.

#### 2.7.6. Sample Size Calculation

The sample size was determined to be 170 participants utilizing the Epi-Info 7.1 statistical software. This calculation was based on a confidence interval (CI) of 95%, a statistical power of 80%, and an assumed approximate proportion of exposure among the control group of 25%. A lack of data regarding serum levels of AAs complicated the calculations. The odds ratio (OR) was hypothesized to be 2.5, with a case-to-control ratio of 1:1.

#### 2.7.7. Quantitative Variables

The status of prediabetes, HT, hereditary predisposition to CVDs, and lipid profile parameters, including TC, LDL-C, HDL-C, and TG, were designated as binary variables based on NCEP ATP III [24]. Smoking status was categorized into three groups: nonsmokers, former smokers, and current smokers. BMI was classified as a ranked variable, with categories of normal weight, overweight, and obesity. Age was stratified into four groups: less than 39 years, 40–49 years, 50–59 years, and older than 60 years. AAs were treated as continuous variables. Preliminary statistical analyses were conducted to assess the normality of all continuous variables. For those variables exhibiting a highly skewed distribution to the right, a log transformation was applied, and if provided, the proportion of zero values was less than 2%.

#### 2.7.8. Statistical Methods

Statistical analysis was conducted using STATA Statistical Software, release 15, College Station, TX, USA; StataCorp LLC. Continuous variables were reported as means ± standard deviation (SD) if they followed a normal distribution, while categorical variables were presented as proportions expressed in percentages. For the primary analysis, a chi-squared test and odds ratios (ORs) and Mantel-Haenszel ORs with 95% confidence intervals (CI) were calculated to examine the associations between categorical or continuous risk factors—like BMI, lipid profile parameters (LDL-C, HDL-C, and TGs), HT, age, and gender—and the primary outcome of interest. The final statistical analysis employed a stepwise method with forward selection in a multiple binary logistic regression model, which included covariates demonstrating significance for the fitted final model. The adequacy of the model was evaluated through a likelihood ratio test (LRT). A receiver operating characteristic (ROC) analysis was performed to determine the area under the curve (AUC) selected for the final logistic regression (LR) model, which targeted amino acids, specifically Alanine and Valine. Cut-off levels for these amino acids were established, and their corresponding sensitivity and specificity for fitted LR models were determined.

## 3. Results

### 3.1. Descriptive Data

In total, data from 75 Kazakh males and 92 females were included in the final analysis. There were 112 individuals with prediabetes and 55 without prediabetes. Only a quarter of participants had a healthy weight, and almost half met the criteria for MS. Baseline characteristics and the distribution of the potential risk factors for prediabetes are summarized in Table 1.

In a comparative analysis, individuals diagnosed with prediabetes exhibited a higher prevalence of unhealthy weight, smoking habits, hypertension, and dyslipidemia relative to the control subjects. The study identified that 70.83% males and 64.21% females were classified as prediabetic. Among the female participants, the majority were non-smokers (85.26%), while nearly half of the male participants (44.44%) also refrained from smoking. Conversely, 8.42% of females and 36.11% of males were identified as current smokers, with 6.32% of females and 19.44% of males classified as former smokers (*p* = 0.0001). Notably, 76% of participants with confirmed hypertension were receiving regular antihypertensive therapy. There was strong evidence of a linear trend, with the proportion of cases with prediabetes increasing with age (*p* = 0.005).

### 3.2. Amino Acids and the Risk of Prediabetes

We analyzed the association between AAs and the risk of prediabetes, as detailed in Table 2. Only Alanine and Valine exhibited significant associations with the risk of prediabetes among the 13 AAs examined. Alanine and valine were found to have an inverse relationship with the risk of prediabetes, indicating that higher serum levels of these AAs corresponded to a reduced likelihood of developing this condition.

**Figure 2:**
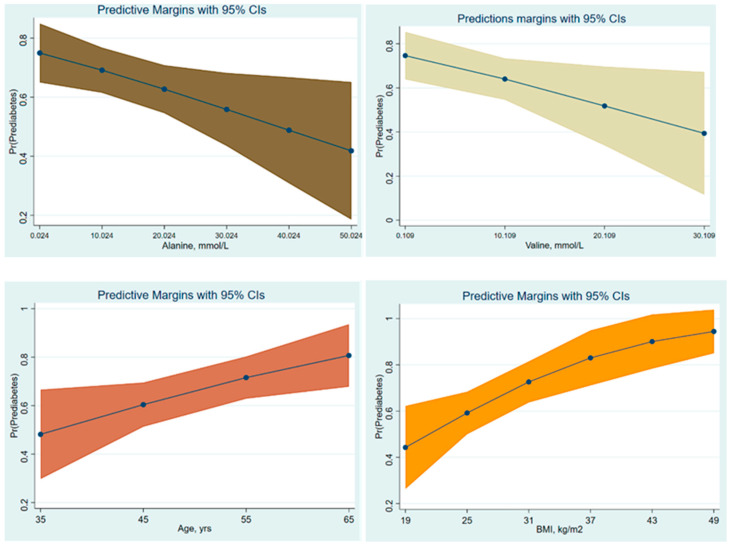
Separated predictive margins of Alanine, Valine, age, and BMI for prediabetes in binary LR models.

We adjusted all AAs for potential confounding factors to elucidate the true associations between AAs and prediabetes risk, which showed that just Alanine and Valine amongst all targeted AAs were associated with the risk of prediabetes, as summarized in Table 3 and Figure 2 The analysis revealed that only age and BMI served as significant confounding factors influencing the relationships between Alanine and Valine, and the risk of prediabetes. At fixed values of covariates in the final binary logistic regression models, Alanine and Valine were significantly inversely associated with the odds of prediabetes. Following the implementation of a stepwise forward selection procedure for covariates within the multiple logistic regression models, the analysis revealed that only age and BMI emerged as significant predictors in the final models, at *p* = 0.033 and 0.049, respectively. Other variables, including smoking status, gender, lipid profile, and history of hypertension, did not significantly impact these associations when they were included step-by-step in the LR models. Notably, when controlling for age and BMI, the strength of the association was enhanced for both Alanine and Valine. Furthermore, the LRT substantiated the significance of both age and BMI across all LR models.

### 3.3. AAs and Prediction of Prediabetes

**Figure 3:**
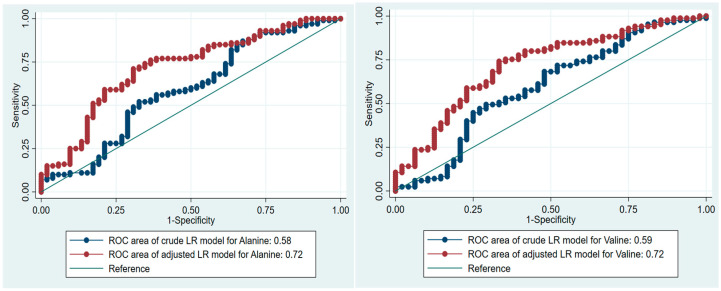
ROC areas of LR model for Alanine and Valine in the prediction of prediabetes, crude and adjusted for age and BMI.

To evaluate the predictive potential of Alanine and Valine, in prediabetes, we conducted an ROC analysis utilizing crude and fitted logistic regression models. These models assessed the associations between AAs and prediabetes adjusting for potential confounding variables, specifically age and BMI (see Table 4 and Figure 3). Our analysis revealed that the inclusion of age and BMI significantly enhanced the predictive accuracy of Alanine and Valine, with the area under the ROC curve (AUC) increasing from 0.57 to 0.73 and 0.60 to 0.72 for simple and multivariable binary logistic regression models, including significant covariates like age and BMI (Figure 3). Furthermore, we determined the optimal cut-off levels for Alanine and Valine with the sensitivity and specificity of adjusted LR models.

## 4. Discussion

In the present study, we investigated the association of 13 AAs with the risk of prediabetes within the Kazakh population. Our findings indicate a significant relationship between Alanine and Valine and the risk of prediabetes. Specifically, Alanine and Valine exhibited negative associations with prediabetes risk. Conversely, the remaining AAs—Tyrosine, Leucine, Isoleucine, Proline, Serine, Threonine, Methionine, Lysine, Aspartic acid, Glutamic acid, and Phenylalanine—were not associated with prediabetes risk.

Emerging evidence suggests that dysregulated metabolism of AAs plays a crucial role in the mechanisms underlying altered gluconeogenesis and the pathogenesis of IR [25,26]. Disturbances in the metabolomic profile are evident in both prediabetes and T2DM [27], with most existing research focusing on the associations between AAs and clinically manifested T2DM, while even fewer studies have addressed prediabetes specifically. Notably, BCAAs—including valine, leucine, and isoleucine—have been identified as potential biomarkers for the early detection of prediabetes and IR and the subsequent risk of T2DM [9], which is confirmed in our study for Valine only. A recent meta-analysis encompassing 22 studies, including 10 studies specifically examining prediabetes and AAs, revealed that tryptophan and BCAAs are directly associated with prediabetes and may serve as predictors of this condition [28,29,30]. In contrast, Owei et al., investigating fasting levels of AAs in individuals with prediabetes, found no significant differences in BCAAs between progressors to prediabetes and non-progressors [31]. These findings are consistent with our results concerning leucine and isoleucine levels. Almost half of the participants in our study group were aged between 50 and 65 years. Previous research indicates that age-related declines in mitochondrial function may impact the metabolic kinetics of AAs synthesis and degradation [32]. Diminished levels of BCAAs in older adults are indicative of an overall catabolic state and a decline in physiological reserve [33]. BCAAs are known to stimulate protein synthesis, while insulin acts to inhibit protein degradation. Consequently, a loss of insulin action may result in the disinhibition of protein breakdown in skeletal muscle, thereby leading to an increased rate of BCAA disappearance [34].

Moreover, aging is associated with a distinctive shift in gut microbiota composition [35]. Gut bacterial proteins exhibit a higher ratio of BCAAs to other amino acids relative to those synthesized by mammalian organisms [36]. Bacteria synthesize BCAAs through conserved pathways that are present in fungi and plants, but absent in mammals [37]. Notably, the expression of BCAAs has been found to be significantly downregulated during aging across multiple species [38]. It has often been observed that advanced age correlates with diminished blood BCAA levels in humans [39,40].

Additionally, ethnic differences in diabetes complications may be influenced by various factors, including BCAA metabolism. For example, South Asian populations tend to experience more severe hyperglycemia, whereas other ethnic groups may exhibit different risk profiles for diabetes-related complications [41]. Recent data have also reported lower levels of BCAAs in individuals who are more physically active [42]. BCAAs play a vital role in the metabolism of brown adipose tissue (BAT) [43]. Yoneshiro et al. observed that cold exposure for two hours preferentially decreased BCAA plasma levels in participants with high BAT activity, suggesting a potential link between BAT functionality and BCAA metabolism [44].

Interestingly, our study identified a lower serum level of Valine and Alanine as a significant predictor of prediabetes risk, a finding not previously reported in the context of prediabetes.

However, a recent systematic review highlighted Alanine and other AAs’ strong positive predictive value in relation to T2DM risk [45]. One study reported reduced levels of Alanine in patients with T2DM [46]. Our data further corroborate the notion that lower levels of Alanine are associated with an increased risk of prediabetes. It has been suggested that lower Alanine levels in obese individuals may be associated with reduced Anaplerotic Oxaloacetate formation and, hence, reduced efficiency of the tricarboxylic acid (TCA) cycle activity and reduced gluconeogenesis. Hepatic glucose production occurs through three primary pathways: glycogenolysis, gluconeogenesis from glycerol, and gluconeogenesis from lactate/pyruvate/AAs [47], with the latter being the predominant pathway during prolonged fasting, which is often disrupted [48,49] in the diabetic liver [50].

Alanine emerges as the principal amino acid catabolized by the liver in all mammals, and all of these amino acids contribute to gluconeogenesis [51,52,53]. In muscle cells, pyruvate is converted to Alanine through the catalytic action of alanine aminotransferase (alanine transaminase, ALT) [54]. Alanine transports amino groups to the liver, where Alanine is subsequently reconverted to pyruvate, enabling its entry into gluconeogenic pathways and to provide energy during fasting or intense exercise [55]. Alanine undergoes transamination reactions by transferring its amino group to form pyruvate and glutamate [56]. Such processes serve to interconnect amino acid metabolism with the citric acid cycle and the subsequent energy production in the liver, kidneys, and small intestine [57].

For gluconeogenesis to occur, Alanine must be converted to pyruvate. This conversion is facilitated by two enzymes, ALT1 and ALT2, which are highly expressed in the liver. ALT1 is found in the cytosol, while ALT2 is located in the mitochondrial matrix [57]. While transamination can occur in the cytosol, recent investigations suggest that mitochondrial ALT2 plays a critical role in gluconeogenesis by facilitating the conversion of Alanine to pyruvate within the mitochondrial matrix [58,59].

Increased expression of ALT2 has been observed in obese humans and murine models [60]. ALT2 expression is regulated by activating transcription factor 4 (ATF4) in the liver. Notably, silencing ALT2 in the liver of obese mice has been reported to diminish alanine-induced hyperglycemia while concurrently elevating serum alanine levels, suggesting a regulatory feedback mechanism pertinent to alanine metabolism in obesity [61].

**Figure 4:**
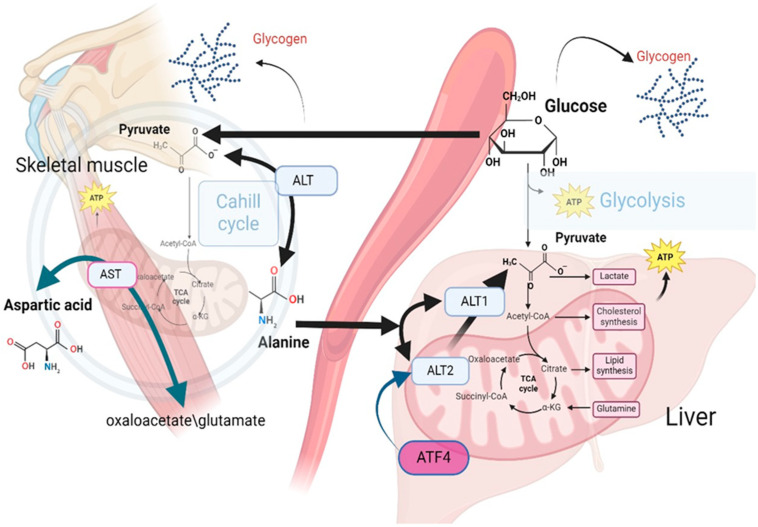
Impact of mitochondrial metabolic activity on circulating levels of Alanine: insights from the Cahill Cycle.

ALT catalyzes the conversion of pyruvate to Alanine in muscle cells. This transamination reaction facilitates the conversion of Alanine and α-ketoglutarate into pyruvate and glutamate [62]. Alanine serves as a vehicle for the transport of amino groups from peripheral tissues to the liver, where Alanine is reconverted to pyruvate, facilitating its subsequent utilization in gluconeogenesis. This pathway is particularly vital during periods of fasting or intense physical exercise. While ALT is predominantly found in the liver, it is also present in other tissues, including the kidneys, heart, and muscle cells, highlighting its importance in amino acid metabolism across different physiological contexts [63] (Figure 4).

The figure illustrates the role of Alanine in gluconeogenesis, emphasizing the enzymatic conversion of Alanine to pyruvate through the action of ALT isoenzymes, specifically ALT1 in the cytosol and ALT2 in the mitochondria. The involvement of ALT2 in this metabolic pathway is critical for gluconeogenesis. Elevated expression levels of ALT2 have been observed in both obese human subjects and relevant animal models, which suggests a potential link between ALT2 activity and obesity-related metabolic alterations. The regulation of ALT2 expression by the transcription factor ATF4 underscores its significant role in the metabolic adaptation in obesity. Experimental evidence indicates that silencing ALT2 in the hepatic tissue of obese mice leads to a decrease in alanine-induced hyperglycemia and a concomitant increase in serum alanine levels. These findings suggest a feedback mechanism governing alanine metabolism in obesity.

Our results did not find an association between threonine and the risk of prediabetes, which is consistent with findings from some previous studies. While certain research has suggested a potential role for amino acids like threonine in metabolic regulation, other studies, including ours, have not demonstrated a significant link between threonine levels and prediabetes risk [64,65]. This supports the view that threonine may not play a critical role in the pathophysiology of prediabetes.

External factors and alterations in cellular metabolomes can trigger distinct glycogenesis pathways, resulting in changes in metabolite utilization. Mitochondrial aging is characterized by reduced TCA cycle efficiency and gluconeogenesis. Mitochondrial dysfunction in high-metabolic-rate tissues, such as the brain, liver, and heart, leads to decreased energy capacity and disrupted redox balance. This decline manifests as diminished TCA cycle efficiency due to reduced oxidative capacity, impaired oxidative phosphorylation, and decreased ATP production [66]. Furthermore, compromised glucose transport and TCA cycle function may contribute to increased alanine catabolism to pyruvate [67]. Hepatic insulin signaling can suppress gluconeogenesis while stimulating de novo lipogenesis, further complicating metabolic regulation [53]. The decline in mitochondrial capacity in the liver and adipose tissues significantly impacts glucose and fatty acid metabolism, contributing to age-related metabolic disorders.

In the present study, we attempted to determine the optimal cut-off points for predicting prediabetes after controlling for potential confounding factors like age and BMI in the Kazakh population. We estimated that the optimal cut-off level for Alanine is 6.23 mmol/L (sensitivity 86%, specificity 30.77%), and for Valine, 2.267 mmol/L (sensitivity 84.71, specificity 41.67%).

### Limitations

This study has limitations that warrant careful consideration:

1. The diagnosis of prediabetes in this study was exclusively based on fasting glucose and HbA1c levels, representing only a subset of potential diagnostic criteria. Incorporating the glucose tolerance test (GTT) could enhance the precision and accuracy of the classification of prediabetes within our cohort.
2. About 75% of the study population consisted of individuals who were either overweight or obese. In this context, HOMA-IR and serum insulin levels, which were not specifically assessed in this study, could represent potential confounding factors in the observed association between AAs and the risk of prediabetes.
3. The prevalence of obesity was approximately double in the case group compared to the control group, with 16.36% of controls classified as obese versus 39.45% of cases. However, the distribution of overweight individuals between the comparison groups was relatively comparable, with 41.82% in the control group and 42.20% in the case group. This similarity in overweight prevalence may help elucidate the lack of observed differences in BCAAs between comparison groups.
4. The imbalance in the ratio of cases to controls presents a concern for diminished statistical power. To mitigate this issue, we increased the total sample size; however, this adjustment may not have entirely offset the potential limitations imposed by the original case–control ratio.
5. The findings of this study are primarily applicable to populations of Kazakh ethnicity, thereby limiting the generalizability of the results to broader populations. As such, caution should be exercised when attempting to extrapolate these findings with other ethnic groups.
6. Not all common 20 AAs were included in this study. Two AAs were excluded from the study. Glutamine exclusion was executed to maintain the study’s limited scope, while concentrating on the metabolic processes linked to the studied demographic’s high-protein, high-fat dietary patterns. Alanine and glutamine are the principal glucogenic amino acids, but Alanine plays a significant role during early-starvation, exposure to high-fat and high-protein diets, and diabetes [60,61,68,69]. Most research concerning the utilization of Alanine and glutamine in gluconeogenesis concludes that Alanine serves as the predominant AA for gluconeogenesis within the hepatic system. In contrast, glutamine assumes a principal role in the renal system and the small intestine [70,71,72]. Tryptophan was omitted because the baseline concentration of tryptophan in the bloodstream of healthy individuals may vary by a factor of five, and this particular AA does not exert a substantial influence on the mechanisms related to MS [46,73]. The detection of AAs in serum utilizing gas chromatography–mass spectrometry (GC–MS) presents notable challenges attributable to the chemical properties of amino acids and the inherent limitations of the GC–MS methodology. However, GC–MS offers the advantage of enabling simultaneous and precise quantification of fatty acids in conjunction with amino acids. This capability is particularly significant in the context of metabolic research.

## 5. Conclusions

Lower serum levels of Alanine and Valine were significantly associated with an increased risk of prediabetes, particularly after controlling for age and BMI in the Kazakh population. These findings suggest a potential metabolic dysregulation that may contribute to the pathophysiology of prediabetes. However, further studies with substantially larger sample sizes are warranted to validate our conclusions and explore the underlying mechanisms.

## Figures and Tables

**Table 1:** Baseline characteristics of the study population.

Characteristics	Number (%)	Prediabetes, %		*p*-Value
		Yes	No	
Gender, male	75 (44.12)	70.83	29.17	0.367
Age (years), mean (SD)	50.55 (7.47)	51.56 (7.45)	48.35 (7.07)	0.008
≤39	10 (5.88)	4.46	9.09	0.04
40–49	70 (41.18)	35.71	52.73	
50–59	70 (41.18)	44.64	32.73	
≥60	20 (11.76)	15.18	5.45	
BMI categories (kg/m^2^)				
≤24.9	43 (25.75)	18.35	41.82	0.001, 0.0002 *
25–29.9	70 (41.92)	42.20	41.82	
≥30.0	54 (32.34)	39.45	16.36	
WC, cm, mean (SD)	97.57(13.29)	99.96 (13.60)	92.75 (11.71)	0.001
Hypertension	50 (29.94)	33.94	21.82	0.109
Antihypertensive therapy	37 (22.56)	78.38	21.62	0.08
Pulse, beats per minute, mean (SD)	74.22 (8.82)	73.83 (8.53)	74.65 (9.36)	0.57
Smoking				
No	113 (67.66)	69.72	63.64	0.03
Quit	34 (20.36)	15.60	30.91	
Yes	20 (11.98)	14.68	5.45	
High fasting glucose (≥5.6)	23 (13.61)	73.91	26.09	0.452
Lipid profile (mmol/L)				
High LDL-C (≥3.3)	73 (42.94)	70.83	29.17	0.367
Low HDL-C (<1.03 in males, <1.29 in females)	57 (33.53)	75.44	24.56	0.097
High TG (≥1.7)	46 (27.06)	79.55	20.45	0.04
MS	82 (50.31)	66.67	18.18	0.0001
Creatinine, mcmol/L, mean (SD)	76.22 (24.53)	74.28 (23.54)	77.18 (19.62)	0.43

* *p* for a trend of odds; BMI, body mass index; WC, waist circumference; MS, metabolic syndrome; LDL-C, low-density lipoprotein cholesterol; HDL-C, high-density lipoprotein cholesterol; TG, triglycerides. Prediabetes and control subjects were compared using a chi-square test for categorical variables and Student test for continuous variables.

**Table 2:** Association between AAs and prediabetes *.

AAs, mmol/L	OR_crude_	95% CI	*p*-Value
Lysine	0.98	0.94; 1.05	0.72
Tyrosine	0.99	0.98; 1.00	0.22
Alanine	0.96	0.94; 0.99	0.019
Valine	0.94	0.89; 0.99	0.015
Leucine	1.02	0.99; 1.05	0.26
Isoleucine	1.01	0.98; 1.04	0.55
Proline	1.01	0.98; 1.03	0.53
Serine	0.96	0.8; 1.16	0.70
Threonine	1.03	0.82; 1.28	0.82
Methionine	1.09	0.95; 1.25	0.23
Aspartic	0.96	0.88; 1.05	0.38
Glutamic	0.92	0.74; 1.14	0.43
Phenylalanine	0.95	0.85; 1.07	0.40

* Binary logistic regression.

**Table 3:** Crude and adjusted ORs of prediabetes for serum level of Alanine and Valine *.

Number of Observations	OR of Prediabetes	95% CI	*p*-Value	Adjusted for	Model
Alanine mmol/L					
156	0.96	0.94; 0.99	0.019	Crude	1
156	0.97	0.94; 0.99	0.022	Age ^α^	2
153	0.97	0.94; 0.99	0.033	Age + BMI ^β^	3
Valine, mmol/L					
136	0.94	0.90; 0.99	0.015	Crude	4
136	0.94	0.90; 0.99	0.014	Age ^α^	5
133	0.95	0.91; 0.99	0.049	Age + BMI ^β^	6

* Multiple binary logistic regression. ^α,β^—LRT (Likelihood Ratio Test) for the significance of age and BMI, *p* < 0.01. Differences in the number of observations are explained by missing data.

**Table 4:** Crude and adjusted AUCs for LR models of association between AAs and prediabetes risk.

LR Models	AUC, 95% CI		Cut-off Point, mmol/L	Sensitivity of the Adjusted LR Models, %	Specificity of the Adjusted LR Models, %
	Crude	Adjusted for Age and BMI			
Alanine	0.58 (0.49; 0.69)	0.72 (0.63; 0.81)	6.234	86.00	30.77
Valine	0.60 (0.48; 0.70)	0.72 (0.63; 0.81)	2.267	84.71	41.67

## Data Availability

The raw data supporting the conclusions of this article will be made available by the authors on request.
